# Generation of human-induced pluripotent-stem-cell-derived cortical neurons for high-throughput imaging of neurite morphology and neuron maturation

**DOI:** 10.1016/j.xpro.2023.102325

**Published:** 2023-06-09

**Authors:** Gautam Wali, Yan Li, Dad Abu-Bonsrah, Deniz Kirik, Clare L. Parish, Carolyn M. Sue

**Affiliations:** 1 Neuroscience Research Australia and University of New South Wales, Sydney, NSW 2031, Australia; 2 Kolling Institute for Medical Research and Department of Medicine, University of Sydney, Sydney, NSW 2065, Australia; 3 Northern Clinical School, Sydney Medical School, University of Sydney, Sydney, NSW 2065, Australia; 4 The Florey Institute of Neuroscience and Mental Health, The University of Melbourne, Parkville, VIC 3052, Australia; 5 Department of Paediatrics, The University of Melbourne, Parkville, VIC 3052, Australia; 6 BRAINS Unit, BMC D11, Lund University, 22184 Lund, Sweden; 7 Honorary Professorship at School of Medical Sciences, Faculty of Medicine and Health, The University of Sydney, Sydney, NSW 2006, Australia; 8 Aligning Science Across Parkinson’s (ASAP) Collaborative Research Network, Chevy Chase, MD 20815, USA

**Keywords:** Cell Biology, Cell culture, Cell Differentiation, Microscopy, Neuroscience, Stem Cells

## Abstract

High-throughput imaging allows *in vitro* assessment of neuron morphology for screening populations under developmental, homeostatic, and/or disease conditions. Here, we present a protocol to differentiate cryopreserved human cortical neuronal progenitors into mature cortical neurons for high-throughput imaging analysis. We describe the use of a notch signaling inhibitor to generate homogeneous neuronal populations at densities amenable to individual neurite identification. We detail neurite morphology assessment via measuring multiple parameters including neurite length, branches, roots, segments and extremities, and neuron maturation.

## Highlights

• Differentiation of cryopreserved cortical neuronal progenitors to mature neurons
• Notch signaling inhibition results in high proportion of mature cortical neurons
• Generate cultures ideal for segmentation of individual neurites for image analysis
• Measure neurite parameters - length, branches, roots, segments, and extremities

## Abstract (editor highlights)

Publisher’s note: Undertaking any experimental protocol requires adherence to local institutional guidelines for laboratory safety and ethics.

## Graphical abstract

**Figure:**
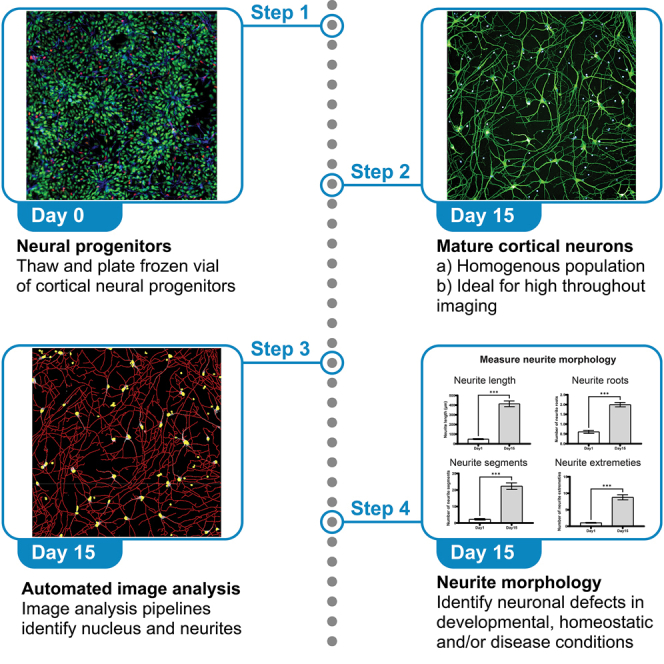

## Before you begin

The pluripotent stem cell-derived cortical neural progenitors used in the following protocol were generated according to the methods described by us^1^ and others.^2^

***Note:*** The cortical neural progenitors and mature neurons should be cultured in a humidified 37°C incubator with 5% CO_2_.

***Note:*** All procedures should be performed in a sterile environment. All waste materials should be considered potentially biohazardous and disposed according to the laboratory’s waste disposal policy.

### Institutional permission

The experiments involving human iPS cells were approved by the Northern Sydney Local Health District Human Research Ethics Committee, Australia (Reference number: RESP/15/314).

### Coating cell culture plate

**Timing: 5 h**

1. Coat the cell culture plates (preferably 96-well plates) with two extracellular matrix proteins, poly-L-ornithine and laminin.

**Table 1:** Poly-L-ornithine dilution for final concentration of 15 μL/mL

Plate	0.01% PLO (μL)	PBS (−/−)	Per well (μL)
6-well plate	150	850	1000
48-well plate	45	255	300
96-well plate	15	85	100

**Table 2:** Mouse laminin dilution for final concentration of 10 μg/mL

Plate	1 mg/mL mouse laminin (μL)	PBS (−/−)	Per well (μL)
6-well plate	10	990	1000
48-well plate	3	297	300
96-well plate	1	99	100

***Note:*** We used 96-well plates as they are useful to culture and compare multiple cell lines and culture conditions (such as drug treatments) in a single plate, thus avoiding plate-to-plate variations. However, this protocol can be adapted to other plate formats. Please refer to Tables 1 and 2 for volumes to be used for plates with 6 and 48 wells.

***Alternatives:*** We have tested and validated the use of extracellular matrix proteins laminin and poly-L-lysine for neuronal differentiation. Alternatively, fibronectin along with poly-D-lysine can be used.

2. Prepare coating solutions as outlined in the materials and equipment section below in Tables 1 and 2.
3. First, coat the culture plate with poly-L-ornithine.
  a. Cover the surface of plate with 100 μL of poly-L-ornithine solution (15 μg/mL, Table 1) for 2 h at room temperature (20°C–25°C).
  b. Aspirate the solution and wash twice with Dulbecco’s phosphate buffered saline without magnesium or calcium (DPBS −/−).

***Alternatives:*** Alternatively, plates with poly-L-ornithine can be left inside the cell culture hood overnight (16–18h) at room temperature (20°C–25°C). The coated plate should be sealed with parafilm to avoid poly-L-ornithine evaporation.

4. Following poly-L-ornithine coating, perform a second coating with mouse laminin.
  a. Cover the surface of the plate with 100 μL of mouse laminin (10 μg/mL, Table 2) for 2 h at room temperature (20°C–25°C).
    ***Note:*** Mouse laminin stock stored at −20°C should be thawed overnight (16–18h) at 4°C before use.
    **CRITICAL:** Mouse laminin should be diluted in DPBS (+/+), diluting in DPBS (−/−) will result in poor attachment and survival.
    ***Alternatives:*** Human laminin-521 (10 μg/mL) can also be used for this step.
  b. Do not wash the mouse laminin coating. Aspirate the mouse laminin immediately before adding the cell suspension in culture media to avoid air drying the coated surface.
    **CRITICAL:** Throughout the coating period ensure the coating solution covers the entire surface of the well. Unevenly coated surface area will result in poor attachment and maturation.

### Preparing cortical base media

**Timing: 30 min**

5. Prepare media as outlined in the materials and equipment section Tables 3 and 4.

**Table 3:** Cortical base media composition

Base media	Volume (mL)	Final concentration
DMEM/F12	48.205	-
NBM	48.205	-
B27 (50×)	1	0.5×
N2 (100×)	0.5	0.5×
ITS-A (100×)	0.5	0.5×
NEAA (100×)	0.5	0.5×
GMAX (100×)	0.5	0.5×
Pen/Str (10,000 U/mL)	0.5	50 U/mL
2-merCap (55 mM)	0.09	49.5 μm
Total	100

**Table 4:** Cortical maturation media composition

Maturation media	Volume (mL)	Final concentration
DMEM/F12	47	-
NBM	47	-
B27 (50×)	2	1×
N2 (100×)	1	1×
ITS-A (100×)	1	1×
NEAA (100×)	1	1×
GMAX (100×)	0.5	0.5×
Pen/Str (10,000 U/mL)	0.5	50 U/mL
Total	100

## Key resources table

**Table:** 

REAGENT or RESOURCE	SOURCE	IDENTIFIER
**Antibodies**		

Mouse monoclonal anti-MAP2 (1:1000)	Abcam	Cat#ab254143; RRID: AB_2936822
Rabbit monoclonal anti-TBR1 (1:1000)	Abcam	Cat#ab183032;RRID: AB_2622323
Rat monoclonal anti-CTIP2 (1:1000)	Abcam	Cat#ab18465; RRID: AB_2936859
Goat anti-Mouse IgG (H + L) Highly Cross-Adsorbed Secondary Antibody, Alexa Fluor™ 488 (1:500)	Invitrogen	Cat#A-11029; RRID: AB_2534088
Goat anti-Rabbit IgG (H + L) Cross-Adsorbed Secondary Antibody, Alexa Fluor™ 594 (1:500)	Invitrogen	Cat#A-11012; RRID: AB_2534079
Chicken anti-Rat IgG (H + L) Antibody, Alexa Fluor™ 594 (1:500)	Invitrogen	Cat#A-21471; RRID: AB_2535874

**Chemicals, peptides, and recombinant proteins**		

Ascorbic acid	Sigma-Aldrich	Cat#A4403; CAS: 50-81-7
B-27^TM^Supplement (50×) (B27)	Gibco	Cat#17504044
Brain-derived neurotrophic factor (BDNF)	STEMCELL Technologies	Cat#78005
Dibutyryl cAMP (dcAMP)	STEMCELL Technologies	Cat#73884; CAS: 16980-89-5
DMEM/F12	Gibco	Cat#11320033;
Dimethyl sulfoxide (DMSO)	Sigma-Aldrich	Cat#D2650; CAS: 67-68-5
Glial cell line-derived neurotrophic factor (GDNF)	STEMCELL Technologies	Cat#78058
GlutaMAX Supplement (100×) (GMAX)	Gibco	Cat#35050061
Hoechst 33342	Thermo Scientific	Cat#62249; CAS: 23491-52-3
Insulin-transferrin-selenium-sodium pyruvate (100×) (ITS-A)	Gibco	Cat#51300044
Laminin-mouse (msLam)	Sigma-Aldrich	Cat#L2020; CAS: 6024-85-7
MEM non-essential amino acids (100×) (NEAA)	Gibco	Cat#11140050
2-Mercaptoethanol (merCap)	Gibco	Cat#21985023
N-2 Supplement (100×) (N2)	Gibco	Cat#17502048
N-[N-(3,5-Difluorophenacetyl)]-S-phenylglycine t-butyl ester (DAPT)	Sigma-Aldrich	Cat#5942; CAS:208255-80-5
Neurobasal plus media (NBM)	Gibco	Cat#A3582901
PBS (+/+) (with Calcium/Magnesium)	Gibco	Cat#14040133
PBS (−/−) (no calcium, no magnesium)	Gibco	Cat#14190144
Penicillin-streptomycin (10,000U/mL) (Pen/Str)	Gibco	Cat#15140122
Poly-L-ornithine solution (PLO)	Sigma-Aldrich	Cat#P4957; CAS: 27378-49-0
Trypan blue	Sigma-Aldrich	Cat#T8154; CAS: 72-57-1
Y-27632 (ROCK inhibitor)	STEMCELL	Cat#72304

**Critical commercial assays**		

BD Cytofix/Cytoperm, Fixation/Permeabilization Kit	BD Life Sciences-Biosciences	Cat#554714; RRID: AB_2869008

**Experimental models: Cell lines**		

Cortical neural progenitors – differentiated from human induced pluripotent stem (iPS) cells – passage 5	Laboratory of Clare Perish	RM 3.5

**Deposited data**		

Figure 2 dataset	This study	https://zenodo.org/record/7882382#.ZE_QMHZKibg
Figure 5 dataset	This study	https://zenodo.org/record/7882388#.ZE_PjXZKibg

**Software and algorithms**		

Harmony® high-content analysis software	PerkinElmer, Inc	Harmony 5.1; RRID: SCR_023543; URL: https://www.perkinelmer.com/product/harmony-5-1-office-hh17000012

**Other**		

PhenoPlate™ 96-well plate	PerkinElmer, Inc	Cat#6055700
Countess™ automated cell counter	Invitrogen	Cat#C10227
Phenix Plus High-Content Screening System	PerkinElmer, Inc	Cat# HH14001000
16-bit Andor Zyla 5.5 megapixel sCMOS camera	PerkinElmer, Inc	Part of the Phenix Plus High-Content Screening System

## Materials and equipment

***Note:*** The cortical base and maturation media described in Tables 3 and 4 can be stored up to a week at 4°C.

***Note:*** The growth factor stocks described in Table 5 can be stored up to 2 weeks at −20°C. For long term-storage, the stocks should be stored at -80°C.

**Table 5:** Growth factors-concentration and abbreviation

Growth factor (stock concentration)	Abbreviation	Final concentration
Brain-derived neurotrophic factor (100 μg/mL)	B	40 ng/mL
Glial cell line-derived neurotrophic factor (100 μg/mL)	G	40 ng/mL
Dibutyryl cAMP (4 mM)	D	50 μM
Ascorbic acid (500 μM)	A	200 nM
Laminin-mouse (1 mg/mL)	L	100 ng/mL
γ-secretase complex/notch pathway inhibitor (DAPT)	D	10 μM

## Step-by-step method details

### Seeding cortical neural progenitor cells for maturation

**Timing: 1 h**

This section outlines how to thaw and seed cryopreserved cortical neural progenitors in cell culture plates to differentiate them to mature cortical neurons.

1. Pre-warm cortical base media in a 37°C water bath.
2. To thaw a frozen vial of neural progenitors, remove the frozen vial of cells from storage and quickly thaw cells in a water bath at 37°C by gently swirling the vial for 1–2 min.

***Note:*** Thaw one frozen vial of cells at a time to prevent prolonged exposure to toxic DMSO present in freezing media at higher temperatures.

3. In a 15 mL conical tube, dilute the 1 mL thawed cell suspension slowly with 7 mls of pre-warmed cortical base media supplemented with ROCK inhibitor (Y27632, 10 μM).

***Note:*** Y27632 significantly enhances recovery of neural progenitor cells from cryopreserved stocks.

4. Centrifuge the cell suspension at 300 × *g* for 5 min at room temperature (20°C–25°C).
5. Gently remove the supernatant using a vacuum suction leaving behind an undisturbed cell pellet.
6. Resuspend the cell pellet in 1 mL of cortical base media supplemented with Y27632 (10 μM).

**CRITICAL:** Gentle titration of the cell pellet is critical for optimal cell survival and necessary for homogenous distribution of the cells in solution. This is important to ensure an accurate cell count and an even distribution of cells upon seeding into wells.

7. Perform cell counting using 10 μL of cell suspension using an automated cell counter or a hemocytometer to calculate cell number and prepare the cell suspension at the desired cell density for seeding.

***Note:*** For this protocol, prepare a cell suspension at 100,000 cells/mL media.

***Note:*** Using an automated cell counter system (for example, Invitrogen Countess 3 Automated Cell Counter) can help identify the proportion of live and dead cells while performing cell counting. One freeze-and-thaw cycle is expected to cause about 5%–10% cell death.

8. Seed 100 μL resuspended cell solution with a density of 100,000 cells/mL media into one well of a 96-well plate.

### Maturation of cortical neural progenitors

**Timing: 15 days**

**Figure 1:**
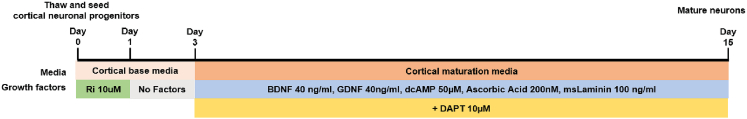
Schematic figure of the protocol to generate highly homogeneous mature cortical neuronal cultures that enables identification of individual neurites – ideal for neurite segmentation essential for neuron morphological assessment

***Note:***Figure 1 outlines the cortical neuron differentiation protocol.

9. At 24 h after cell seeding, using a light microscope confirm cells have survived, attached to the plate, and are evenly distributed within the well.

**CRITICAL:** Low cell densities will have a significant impact on neuron maturation. If the cell cultures have more than 20–25% cell death, then it is best to discard and repeat the experiment.

10. Day 1: remove the cortical base media with Y27632 and gently wash each well once with PBS −/− to remove residual Y27632. Replace media with cortical differentiation base media without Y27632.

***Note:*** On day 1 and 3, perform a 100% media change i.e., replace all 100 μL of media. The media used on these two days are different.

11. Days 3–15: Replace media with cortical maturation media supplemented with growth factors BGDAL (growth factor concentrations and abbreviations and detailed in Table 5) and ϒ-secretase complex/notch pathway inhibitor (DAPT).

**CRITICAL:** the growth factors should be added to the media on the day of media change to minimize degradation.

***Note:*** Media change should be performed every second day. As neurons mature, they are more sensitive to total media aspiration.

***Note:*** From days 5 to 15, perform 90% media change i.e., remove 90 μL media and add 90 μL fresh media. The differentiation media on days 5–15 is the same. So, the 10% left-over media will not impact the concentration of the growth factors.

***Note:*** At the end of step 11, cortical neurons are day 40 of maturation, noting cells are cryopreserved at day 25 after the onset of iPSc cortical differentiation, plus a additional 15 days in culture (according to the aforementioned steps).

### Immunostaining of mature cortical neurons

**Timing: 5 h**

This section outlines how to perform immunostaining^3^ of mature cortical neurons.

***Note:*** A ready to use kit, Cytofix/Cytoperm^TM^Fixation/Permeabilization Kit was used for the fixation, blocking, antibody incubations and wash steps involved in the immunofluorescence staining protocol.

***Note:*** The volume added to each well of the 96-well plate is 100 μL in steps 12 to 26.

12. Aspirate media and wash wells once with PBS−/− to remove residual media. Add 100 μL of Cytofix solution for 25 min at room temperature (20–25).
13. Aspirate Cytofix solution and wash cells twice with PBS−/−.
14. To permeabilize and block the cells for immunostaining, add Cytoperm solution for 30 min at room temperature (20°C–25°C).

**Pause point:** If it is not possible to continue performing further immunostaining steps on the same day, the samples can be stored at 4°C overnight (16–18 h).

15. Prepare 1:1000 diluted primary antibodies in Cytoperm solution.
16. Aspirate the Cytoperm solution from the wells and add the diluted primary antibodies into wells.
17. Incubate at room temperature (20°C–25°C) for 1 h.
18. Wash the cells twice in Cytoperm solution to remove primary antibodies.
19. Prepare 1:500 diluted secondary antibodies in Cytoperm solution.
20. Add the diluted secondary antibodies into wells.
21. Incubate the plate at room temperature (20°C–25°C) for 30 min.
22. Wash the cells twice in Cytoperm solution to remove secondary antibodies.
23. Prepare 1:10,000 diluted Hoechst in Cytoperm solution.
24. Add the diluted Hoechst into wells.
25. Incubate the plate at room temperature (20°C–25°C) for 10 min.
26. Wash the cells twice in Cytoperm solution to remove the residual dyes and then add 100 μL of Cytoperm solution into each well of 96-well plate.

***Note:*** If the samples are not imaged immediately, the plates should be sealed with Parafilm and stored at 4°C. The plates should be protected from light by wrapping with aluminum foil. It is recommended to image the cells within 48 h of immunostaining before the fluorochromes start fading.

### High throughput imaging and analysis of early and mature neurons

**Timing: 2 h**

***Note:*** Here we describe high throughput imaging and analysis performed using the Perkin Elmer PhenixPlus high content screening microscope. Other licensed image analysis software (such as MetaXpress software and Molecular devices), and open source image analysis software (such as Image J) can also be used for assessment of neuron morphology.

27. Image the 96-well plate with neurons on PhenixPlus using a 16-bit Andor Zyla 5.5 megapixel sCMOS camera and a 20× water objective. The imaging was done under a 2× digital binning resulting in image size of 2160 × 2160.
28. Take z-stacked images to capture neurons at different depths. Image analysis is performed on maximum projection images.

***Note:*** The depth of the neuronal cultures can vary in different areas within the same well. Ensure appropriate number of image stacks are captured to image the entire depth of neurons across the well.

**Figure 2:**
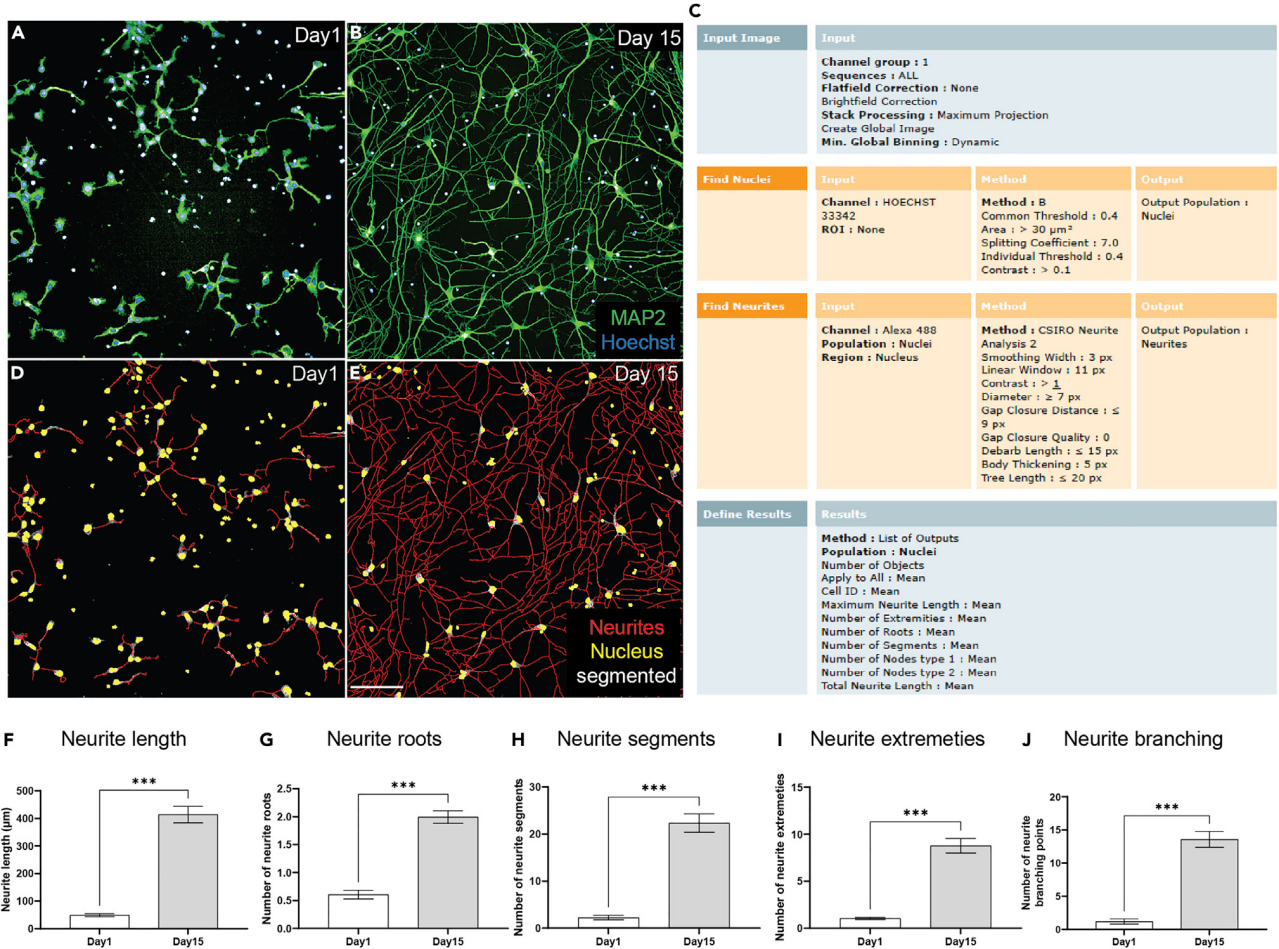
Neurite morphology assessment (A) Early neurons i.e., Day 1 after thawing neural progenitors. (B) Mature neurons i.e., Day 15 after thawing neural progenitors. (C) Harmony (Perkin Elmer) image analysis software-based analysis sequence for automated analysis of neurite morphology. (D and E) shows the identification of nucleus and neurites in early (D) and mature (E) neurons. (F–J) shows that our assay is sensitive to changes in neurite morphology as shown by increased (F) neurite length (G) neurite roots (H) Neurite segments, (I) neurite extremities and (J) neurite branching in Day 15 mature neurons compared to Day1 early neurons. All experiments were performed in duplicate wells. Mean ± SEM. Scale: 100 μm.
  b. Label neurons with Hoechst (to identify nuclei) and MAP2 (to identify MAP2 positive neurites) and image (Figures 2A and 2B).
  c. Analyze images of neurons using image analysis pipeline, detailed in steps 32 and 33 below (Figure 2C).
  d. Segment nuclei and neurites (Figures 2D and 2E) and measure neurite outgrowth parameters (Figures 2F–2J). The dataset for Figures 2F–2J is available at https://doi.org/10.5281/zenodo.7882382.

***Note:*** The Harmony software provides building blocks for image segmentation. Segmentation involved breaking down the image into discrete objects such as individual nuclei or neurites.

***Note:*** Before finding neurites, we segmented the image using the “Find nuclei” building block to identify the nucleus that the neurites extend from.

29. Acquire at least fifteen fields of view per well.
30. Perform image analysis using Harmony, the image analysis software, built in with PhenixPlus high-content imaging microscope.
31. Conduct neurite morphology assessment.
  a. Measure parameters of neurite outgrowth, neurite length, roots, extremities, and segments in early (Day 1 post seeding neural progenitor cells) and mature neurons (Day 15 post seeding neural progenitor cells) (Figure 2).

32. Optimally segment fluorescently labelled nuclei by applying 1 of 4 pre-defined methods in the “Find nuclei” building block.
  a. To choose the best pre-defined method, test all the methods available and choose the one method that is most convincing visually to have accurately selected nuclei.
  b. Test this in multiple different fields of views in multiple wells to ensure the selected method works well across different conditions.

***Note:*** The pre-defined “Find nuclei” functions generally work quite well. However, if needed parameters such as nuclei area, threshold and contrast can be tweaked to suit your images. A cell number dilution series experiment can be setup to test and validate which pre-defined method works well for your cells.

33. Measure parameters of neurite outgrowth including neurite length, branching, extremities, and segments using the “Find neurites” building block.

**Figure 3:**
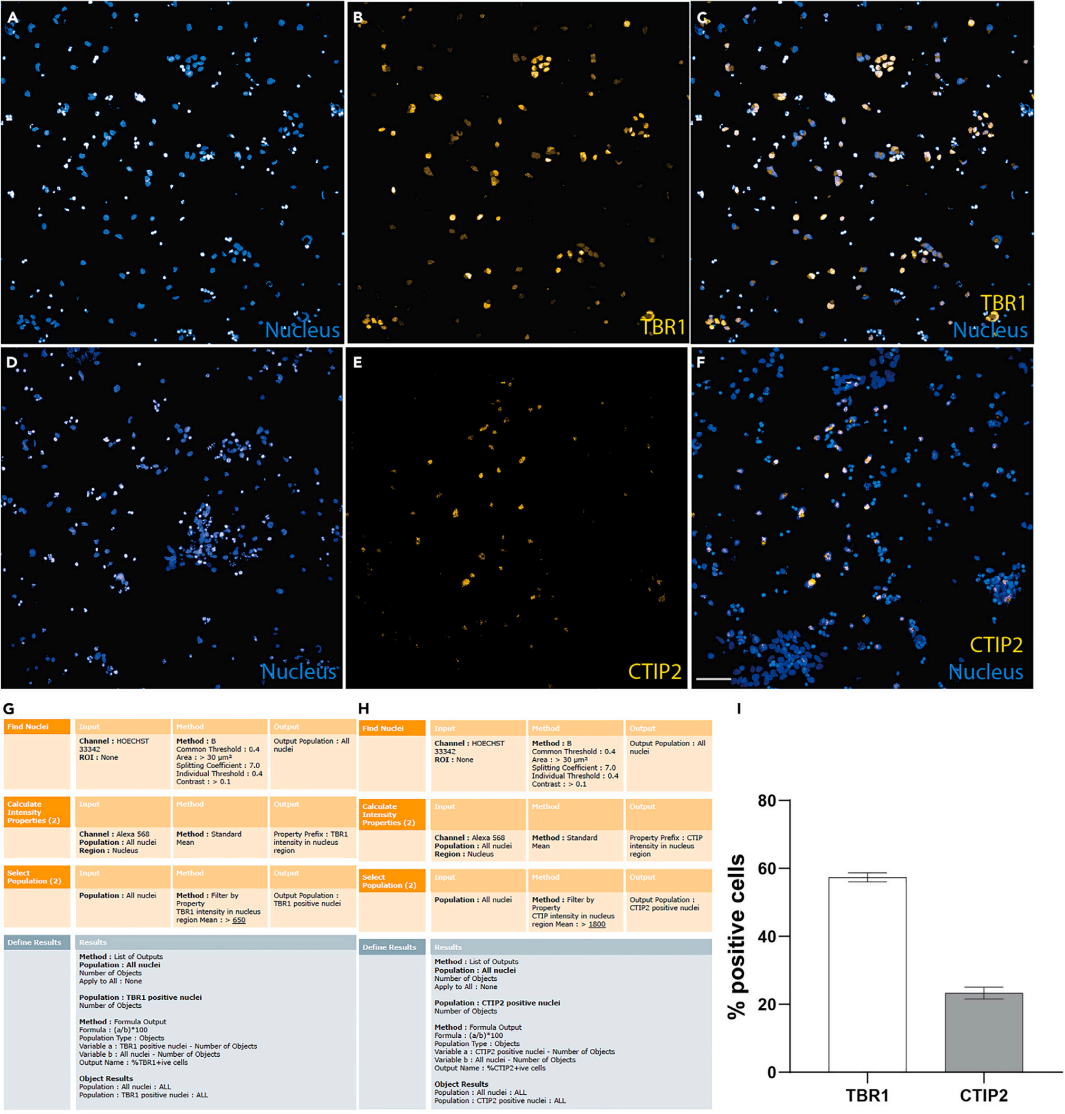
Neuron maturation assessment (A–F) Neurons labelled with Hoechst (A), mature cortical marker TBR1 (B) and merged imaged of Hoechst and TBR1 (C). Neurons labelled with Hoechst (D), mature cortical marker CTIP2 (E) and merged imaged of Hoechst and CTIP2 (F). (G and H) Harmony (Perkin Elmer) image analysis software-based analysis sequence for automated analysis of neuron images to measure the percentage of neurons positive for mature cortical marker TBR1 (G) and CTIP2 (H). Negative isotype control was used to set the threshold to identify positively stained cells. (I) Percentage of neurons positive for TBR1 and CTIP2 cortical neuronal markers. All experiments were performed in duplicate wells. Mean ± SEM. Scale: 100 μm.

***Note:*** The “Find neurites” building block is tailored to detect neurites and provides a set of interactive illustrations and tuning dialogues to visualize the neurite detection results and adjust the parameters if required. Figure 2C shows the parameters we used. We measured the neurite outgrowth parameters for the early (Day1) and mature (Day 15) neurons. As expected, Day 15 mature neurons had relatively longer and more complex MAP2 positive neurites (Figures 2F–2J).

34. Conduct neuronal maturation assessment (mature neurons).
  a. Label neurons with Hoechst (to identify nuclei) and TBR1 or CTIP2 (to identify mature cortical neurons) (Figures 3A–3F).
    ***Note:*** TBR1 and CTIP2 mature cortical markers are expressed in the nucleus^4^ (Figures 3B and 3E).
  b. Analyze the images using image analysis pipeline presented in Figure 3G (for TBR1) and 3H (for CTIP2).
  c. Use the “find nuclei” building block to segment the image and find nuclei (as described in step 32).
  d. Use the “Calculate Intensity Properties” building block to determine the fluorescence intensity of TBR1/CTIP2 markers in the nucleus region.
    ***Note:*** Nucleus expressing the TBR1/CTIP2 markers will have a high fluorescence intensity. Set an intensity threshold using an isotype control to identify positively stained nuclei.
  e. Measure the percentage of TBR1 and CTIP2 positive cells using the formula: (number of TBR1/CTIP2 positive nuclei ÷ total number of Hoechst labelled nuclei) ∗ 100 (Figure 3H).
    ***Note:*** Percentage of other mature cortical markers such BRN2, SATB2 and CUX1 can also be measured using the same image analysis pipeline.

## Expected outcomes

**Figure 4:**
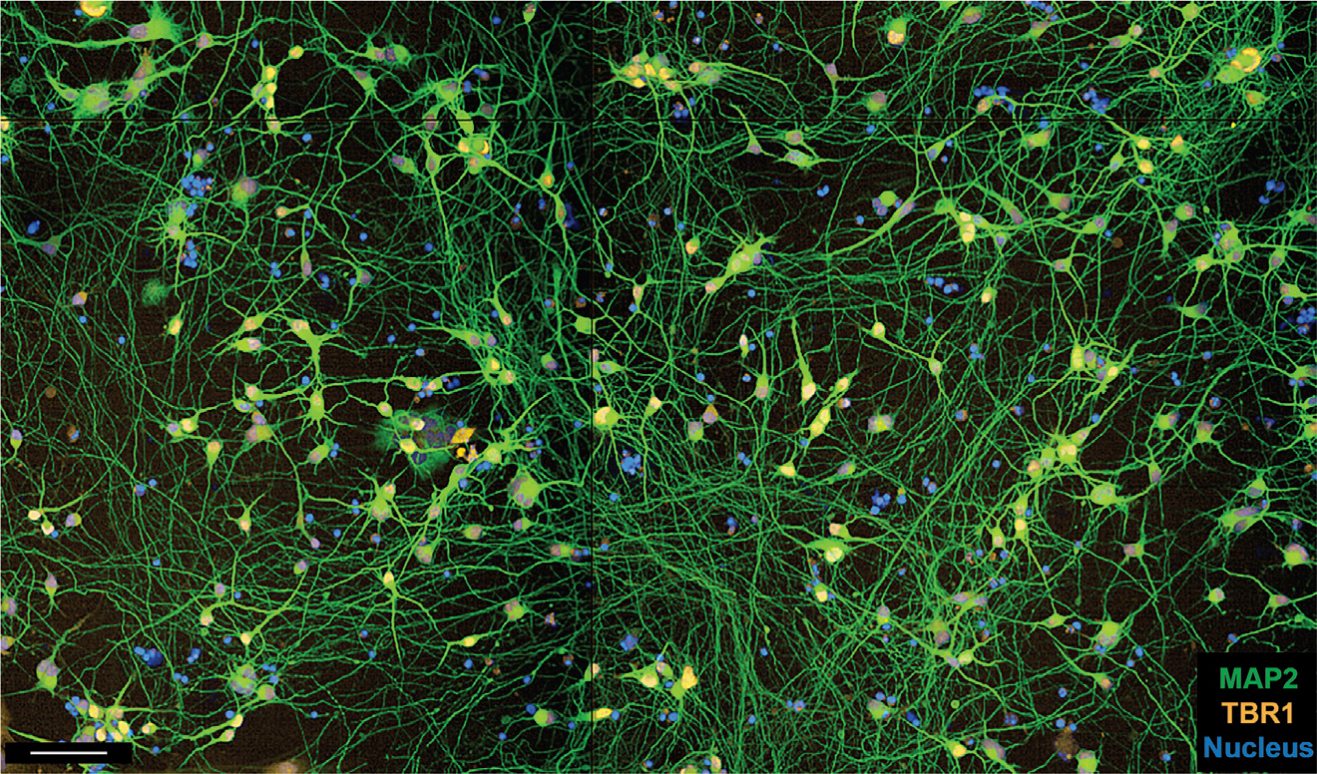
Mature cortical neurons immunostained with TBR1 to identify mature cortical neurons (expressed in the nucleus), MAP2 to identify MAP2 positive neurites and Hoechst to identify nucleus Scale: 100 μm.

The differentiation protocol described above should result in mature cortical neurons (Figure 4). Notch signaling pathway regulates the proliferation of cortical progenitors.^5^ DAPT, an inhibitor of notch signaling, is often used to promote cortical progenitors exit cell-cycle *in vitro* resulting in neuron maturation. Reflective of the impact of prolonged Notch inhibition, our neuronal cultures had high proportion of mature cortical neurons, seen with the expression of mature cortical neuron markers TBR1 and CTIP2. By day 15 after seeding neural progenitors, the neurons formed long and complex neurites (Figures 2 and 4). The neurons were uniformly spread out across the wells with minimum neuronal clustering allowing segmentation of individual neurites for image analysis (Figure 2). These outcomes (homogeneous cortical maturation and uniformly spread-out cultures) are ideal for imaging and analyzing neurite morphology. Although this protocol generates a high population of mature cortical neurons, it is possible that a small population of non-neurons may also be generated. Using cell type specific markers, such as GFAP for astrocytes can help identify any undesired non-neuronal cell types.

**Figure 5:**
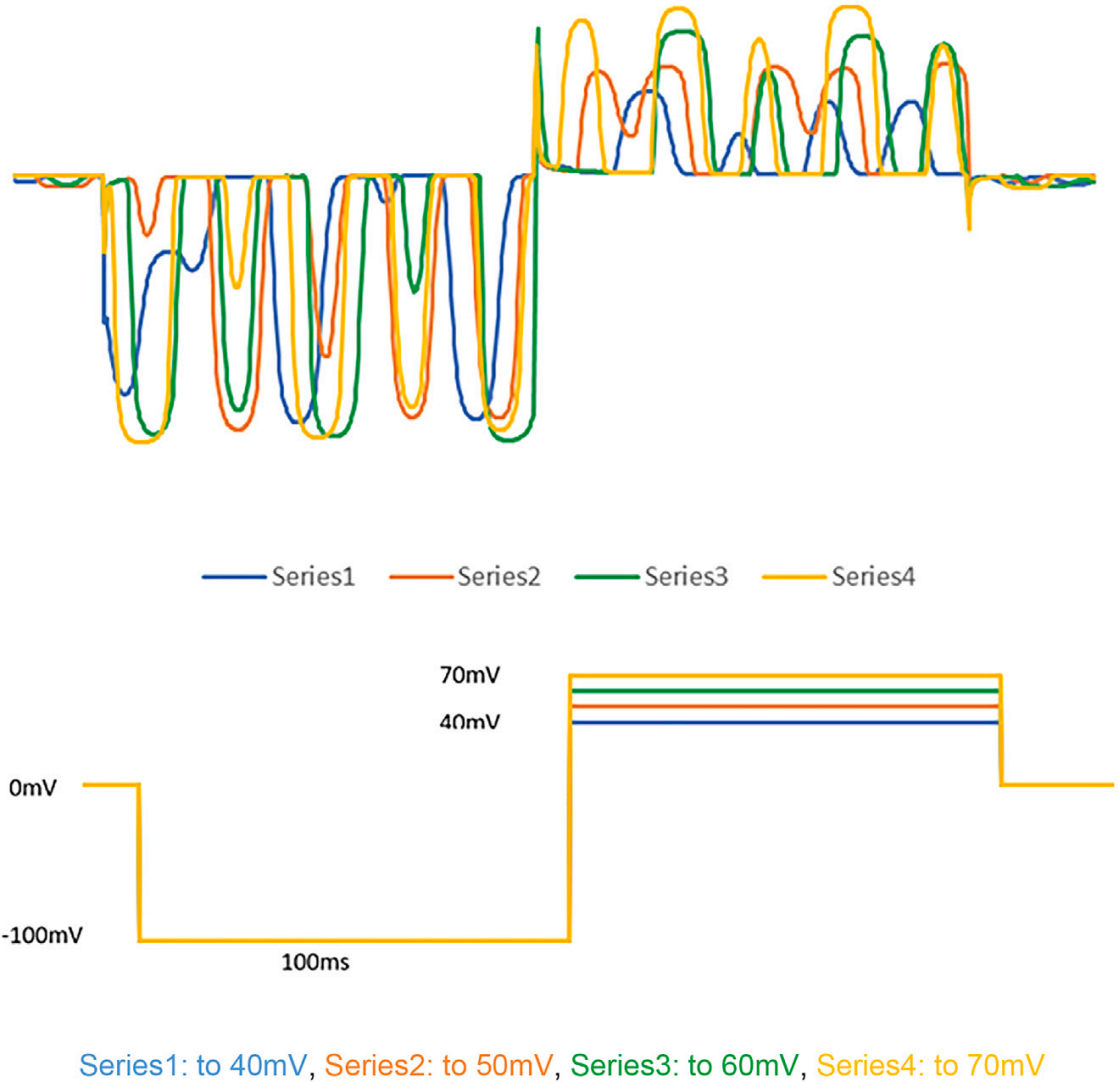
Whole cell patch clamping showed the differentiated neurons are functional

To further test if the neurons are mature, neuron functionality was tested using whole cell patch clamp (Figure 5). The electrophysiological activities of neurons were measured in voltage clamp mode. Voltage pulses are delivered to step the membrane potential from -100 mV to +70 mV (four steps from +40 mV to +70 mv using 10 mV interval steps) and the current responses are recorded. Cells exhibited spontaneous excitatory inward and outward currents indicating the formation of functional excitatory neurons. The dataset for Figure 5 is available at https://doi.org/10.5281/zenodo.7882388.

## Limitations

The image analysis presented in this protocol is performed using Harmony Perkin Elmer, a licensed image analysis software. However, similar image analysis measuring neurite outgrowth can be performed using open source image analysis software’s such as Cell Profiler^6^ and ImageJ with NeuronJ PlugIn.^7^ Both Cell Profiler and ImageJ support multiple image file formats such as JPEG and TIFF.

## Troubleshooting

### Problem 1

Low neural progenitor to mature cortical neuron differentiation efficiency (relevant to steps 9–11).

### Potential solution

The cortical neuron progenitors used in this study were differentiated from iPS cells using a dual SMAD neural induction protocol, as previously described.^1^ The progenitors were then expanded and maintained using FGF2. At day 25, before cryopreserving the cells, we performed immunohistochemistry to confirm the emergence of basally dividing TBR2+ intermediate progenitor cells. Cryopreserving late/matured progenitors will lead to low survival rate during the thawing process. This has been tested in multiple cell lines.

It is essential to follow this iPS to neural progenitors differentiation protocol to successfully differentiate neural progenitors to mature cortical neurons.

### Problem 2

Early and late cell loss during the differentiation protocol (relevant to steps 9–11).

### Potential solution

All differentiation protocols described in this manuscript use the same density of 10,000 per well of a 96-well plate or 0.32 cm^2^ while setting up differentiation. Excessively low cell densities will significantly impact neuron maturation. Setting up replica (sister) plates will allow fixing and counting cells as you progress through the differentiation protocol. For example, our differentiation protocol is for 15 days. We suggest fixing sister plates every 5 days i.e., Day 1, Day 5 and Day 10 to quantify cell numbers.

Case 1. Early cell loss: Measuring cell numbers at Day 1 is informative to understand if the cells seeded on Day 0 are adhering well. Cell loss at this early stage can indicate possible issues with thawing cell vials–keeping vials out for too long and coating plate surface with PLO/laminin–uneven coating surface.

Case 2. Late cell loss: Healthy neuronal cultures have a small proportion of aged apoptotic cells that may be lost during the course of the 15-day differentiation. This is estimated to be 5–10% cell loss. If there is a high cell loss, then this can indicate A) unhealthy neuronal cultures arising from- incorrectly maintained differentiation conditions or lack of differentiation ability of the iPS cell line or pathogenic disease associated effects B) As the neurons differentiate and mature, they make neuronal networks that can lift of relatively easily additional care should be taken to gently change media without disturbing the neuronal networks. We suggest using a motorized multichannel pipette for media change. This allows having low and consistent media suction speed and pressure across all wells of a plate and between plates. As mentioned in the CRITICAL note after step 11, perform a 90% media change (not 100%) to avoid the risk of lifting off neurons from the plate.

### Problem 3

High cell proliferation during the differentiation protocol (relevant to steps 9–11).

### Potential solution

As suggested above, setting up replica (sister) plates will allow fixing and counting cells as you progress through the differentiation protocol. It is well established that DAPT promotes cortical progenitors to exit cell-cycle and promotes neuron maturation. The same has been observed in our differentiation protocol. If the cells continue to proliferate highly after continued prolonged treated with DAPT, it would be advised to recheck DAPT stock preparation and storage conditions.

### Problem 4

Neurons retracting neurites (relevant to steps 9–11).

### Potential solution

We have observed that neurons are highly sensitive to temperature changes. Disturbance by handing of the incubators with neurons should be kept to a minimum. Frequent opening/closing the incubators or having the neuron plate outside the incubator for longer durations can cause neurite retraction.

## Resource availability

### Lead contact

Further information and requests for resources and regents should be directed to and will be fulfilled by the lead contact, Gautam Wali (g.wali@neura.edu.au).

### Materials availability

This study did not generate new unique reagents, cell, or mouse lines.

### Data and code availability

The protocol includes all data generated during the study. Datasets are available at https://doi.org/10.5281/zenodo.7882382 and https://doi.org/10.5281/zenodo.7882388.
